# Differential regulation of diacylglycerol kinase isoform in human failing hearts

**DOI:** 10.1186/1749-8090-6-65

**Published:** 2011-05-08

**Authors:** Olga Bilim, Tetsuro Shishido, Shuji Toyama, Satoshi Suzuki, Toshiki Sasaki, Tatsuro Kitahara, Mitsuaki Sadahiro, Yasuchika Takeishi, Isao Kubota

**Affiliations:** 1 Department of Cardiology, Pulmonology, and Nephrology, Yamagata University School of Medicine, Yamagata, Japan; 2 Department of Cardiovascular, Thoracic, and Pediatric Surgery, Yamagata University School of Medicine, Yamagata, Japan; 3 Department of Cardiology and Hematology, Fukushima Medical University, Fukushima, Japan

## Abstract

Evidence from several studies indicates the importance of Gαq protein-coupled receptor (GPCR) signaling pathway, which includes diacylglycerol (DAG), and protein kinase C, in the development of heart failure. DAG kinase (DGK) acts as an endogenous regulator of GPCR signaling pathway by catalyzing and regulating DAG. Expressions of DGK isoforms α, ε, and ζ in rodent hearts have been detected; however, the expression and alteration of DGK isoforms in a failing human heart has not yet been examined. In this study, we detected mRNA expressions of DGK isoforms γ, η, ε, and ζ in failing human heart samples obtained from patients undergoing cardiovascular surgery with cardiopulmonary bypass. Furthermore, we investigated modulation of DGK isoform expression in these hearts. We found that expressions of DGKη and DGKζ were increased and decreased, respectively, whereas those of DGKγ and DGKε remained unchanged. This is the first report that describes the differential regulation of DGK isoforms in normal and failing human hearts.

## Introduction

Epidemiological studies have suggested that cardiac hypertrophy is an independent risk factor for the development of heart failure and is associated with increased cardiac morbidity and mortality in patients with cardiovascular diseases [1-3]. Recent *in vivo *and i*n vitro *studies have focused on protein kinase signaling cascades as the molecular mechanisms regulating the hypertrophic response of cardiomyocytes [4,5]. Among these signaling pathways, the Gαq protein-coupled receptor (GPCR) signaling pathway, which includes diacylglycerol (DAG) and protein kinase C (PKC), plays a critical role in the development of cardiac hypertrophy and progression to heart failure (HF) [6-8].

The main route for termination of DAG signaling is through phosphorylation by DAG kinase (DGK) to produce phosphatidic acid [9,10]. To date, at least 10 DGK isoforms--DGKα, β, γ, δ, ε, ζ, η, θ, ι, and κ-- have been identified in mammals; DGK isoforms have been reported to be expressed in various tissues, suggesting the importance of these kinases in basic cellular functions [11,12]. In rodent hearts, the expressions of DGKα, ε, and ζ isoforms have been detected, and differential regulation of DGK isozymes in the development of pressure-overload cardiac hypertrophy and in left ventricular remodeling after myocardial infarction has been shown [13,14]. Evidence from several *in vitro *[15] and *in vivo *[16] studies suggests that DGKζ blocks GPCR-induced activation of PKC, and suppresses cardiomyocyte hypertrophy and progression of heart failure.

However, the expression of DGK isoforms in failing human heart has not been previously examined. Therefore, the purpose of this study was (1) to identify the DGK isoforms in the right atrial myocardium in patients undergoing cardiac surgery with cardiopulmonary bypass and (2) to examine changes in expressions of DGK isoforms in cases of failing human heart due to chronic volume overload.

## Materials and methods

### Study patients and materials

Intraoperative samples of the right atrial myocardium were obtained from a total of 17 patients who underwent cardiac surgery at the Yamagata University Hospital between February 2006 and September 2007. All procedures were performed in accordance with the ethical standards outlined in the Declaration of Helsinki of 1975 (revised 1983). The research protocol was approved by the institution's ethical committee, and written informed consent was obtained from all subjects.

Heart samples were obtained from 10 consecutive patients [mean age: mean (SD), 63 (13) years; 7 men and 3 women] admitted for surgical correction of chronic regurgitation associated with mitral valvular lesions (valvular replacement or valvuloplasty, n = 6) or combined dual-valve replacement (n = 4) (Table 1). Right atrial tissue samples collected from 7 patients with aortic dissection, no structural cardiac diseases, and normal heart function were used as controls. Small samples of the right atrium tissue were collected when patients underwent median sternotomy with aortic and right atrial cannulation. The samples were obtained in the operating room and rapidly frozen in liquid nitrogen until further use.

**Table 1:** Demographic and clinical features of patients with heart failure due to volume-overload

				Echocardiographic measurements					Cardiac catheterization data					
				
**Patient No**.	Age (years)/Sex	Diagnosis	NYHA class	LVDd mm	LAD Mm	LVFS %	LVEF %	MR	CI	RAP A/V/M	RVP S/D/E	PAP S/D/M	PCWP A/V/M	LVP S/D/E
1	45/m	MR	III	72	48	40	69	III	2.24	13/13/11	37/6/10	40/17/27	25/37/22	113/8/28
2	42/f	MR TR, ASR,	III	54	44	38	68	II	3.33	13/11/9	34/4/13	30/14/21	23/26/17	128/4/17
3	77/m	MSR, TR	III	57	62	30	56	II	2.72	-/4/3	42/2/9	30/12/19	34/15/12	129/1/5
4	55/m	MR	IV	57	35	40	70	III	2.72	9/7/5	39/3/6	45/19/31	24/35/21	118/-9/27
5	74/f	MR, TR, ASR	III	64	55	23	40	III		-	-	-	-	-
6	65/f	MR, TR	IV	64	80	35	64	IV	2.29	-/18/14	75/14/18	63/26/45	-	-
7	72/m	MR, TR, AR	IV	77	65	48	78	III	2.98	6	35/7	33/15/20	10	150/12
8	63/m	MR, TR	IV	61	57	36	65	III	1.98	3/3/2	18/0/3	15/6/10	9/8/5	102/0/36
9	58/m	MR, TR, ASR	III	70	61	22	43	II	2.42	-/6/4	34/0/6	28/13/21	-/26/16	136/2/21
10	80/m	MR, TR	IV	66	57	23	46	II	1.95	11/12/10	34/8/11	34/19/26	25/30/25	86/8/12

MR, mitral regurgitation; MSR, mitral stenosis and regurgitation; AR, aortic regurgitation; ASR, aortic stenosis and regurgitation; TR, tricuspid regurgitation; LVDd, left ventricular diastolic diameter; LAD, left atrium diameter; LVFS, left ventricular fractional shortening; LVEF, left ventricular ejection fraction; CI, cardiac index (L/min/m^2^); RAP, right atrial pressure; RVP, right ventricular pressure; PAP, pulmonary artery pressure; PCWP, pulmonary capillary wedge pressure; LVP, left ventricular pressure (mmHg); NYHA, New York Heart Association

### RNA preparation and reverse transcription-polymerase chain reaction analysis

Extraction of DNA-free total cardiac RNA was performed using the RNeasy fibrous tissue mini kit (Qiagen, Tokyo, Japan) according to the manufacturer's instructions. For conventional reverse transcriptase polymerase chain reaction (RT-PCR) analysis, 1 μg of total RNA was reverse-transcribed using the QuantiTect reverse transcription kit (Qiagen) [17,18]. The primer pairs for human DGK isoforms used for PCR analysis were designed on the basis of GenBank sequences (DGKα, BC031870; DGKβ, AB018261; DGKγ, BM669549; DGKδ, BC006561; DGKε, U49379; DGKζ, U94905; DGKη, AK098302; DGKθ, BC063801; DGKι, AF061936; DGKκ, AB183864; GAPDH, M33197). PCR products were characterized by performing agarose gel electrophoresis on 2% Tris/borate/EDTA (TBE) agarose gel and visualized by ethidium bromide staining. Densitometry of the bands was performed using ImageJ (v1.29s NIH). The intensities of the bands were normalized for GAPDH. Each reaction included positive and negative controls. Total RNA from Human brain (Ambion, Cat. No. AM7962) and HeLa cells were used as positive controls.

### Statistical Analysis

Data are presented as mean (SD). Differences between the 2 groups were evaluated using Student's *t *test, and a P-value of <0.05 was considered statistically significant. All statistical analyses were performed with a standard statistical program package (JMP version 8; SAS Institute Inc., Cary, North Carolina).

## Results

### Analysis of DGK isoform expression in a normal heart

First, we confirmed the expression of DGKα, β, γ, ε, ζ, η, θ, and ι in human brain cells and that of DGKδ, ε, ζ, and η in the HeLa cell line by using RT-PCR (data not shown). The human brain or HeLa cells did not show the expression of DGKκ. This finding is consistent with that reported by a recent study that showed the presence of DGKκ mRNA only in the human testis and placenta tissues [19]. Therefore, we used mRNA from the human brain and HeLa cells as positive control for further experiments using human heart tissue.

### Clinical and hemodynamic characteristics of patients with heart failure

Clinical characteristics and echocardiographic and hemodynamic measurements of heart failure patients undergoing valvular replacement or valvuloplasty are shown in Table 1. Five patients showed New York Heart Association (NYHA) functional class III heart failure, and another 5 patients showed class IV heart failure. Echocardiography revealed that the patients had marked left ventricular dilatation (LVDD, 64 (7) mm vs. 47 (4) mm in control, P < 0.0001) and left atrium dilatation (LAD, 56 (13) mm vs. 35 (6) mm in control, P = 0.0011). Six patients had atrial fibrillation. Five patients had low cardiac index (<2.5 L/min/m^2^. Elevation of pulmonary arterial pressure (PAP systolic ≥30 mmHg), pulmonary capillary wedge pressure (mean of PCWP ≥12 mmHg), and right ventricular pressure (RVP systolic ≥30 mmHg) was observed in 7, 6, and 8 patients, respectively. Echocardiography revealed moderate to severe tricuspid regurgitation in 4 patients. A large proportion of patients had right atrial overload.

**Figure 1:**
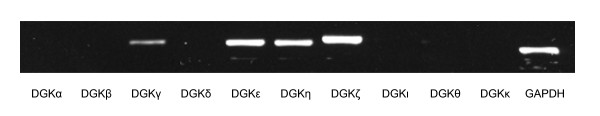
**Expressions of diacylglycerol kinase (DGK) isoforms in normal human right atrium**.

The expression of the DGK isoforms in the human right atrium was examined in the control heart specimens by using RT-PCR. RT-PCR analysis performed using oligonucleotide primers specific for the 10 human DGK isoforms revealed 4 DGK isoforms DGKγ, DGKη, DGKε, and DGKζ in normal human hearts (Figure 1).

### Changes in DGK isoform expression in hearts with volume-overload

**Figure 2:**
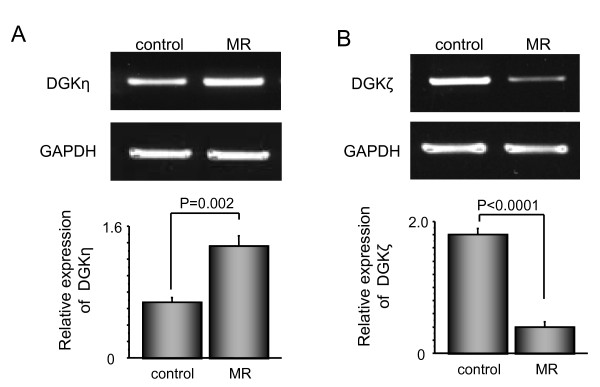
**Diacylglycerol kinase (DGK)η (A) and DGKζ (B) mRNA expressions in right atrial samples obtained from patients with and without heart failure**.

To investigate the changes in mRNA levels of the DGK isoforms in patients with volume-overloaded atria, we examined the expression levels of DGKγ, DGKη, DGKε, and DGKζ isoforms in the right atrium specimens obtained from heart failure patients and compared them with the corresponding expression levels in the control heart samples. Volume overload caused changes in the expression levels of DGKη and DGKζ. Expression level of DGKη was significantly increased (Figure 2A), while that of DGKζ was significantly decreased (Figure 2B). In contrast, expression levels of DGKγ and DGKε remained unchanged in the patients with chronic overload in the right atrium.

## Discussion

All DGK family members share conserved domains and are subdivided into 5 functional classes on the basis of the subtype-specific regulatory domains [12]. DGK represents a large family of isoforms that differ remarkably in their structure, tissue expression, and enzymatic properties, and are encoded by different genes [11]; however, to the best of our knowledge, DGK isoform expression in the human heart has not been previously examined.

In the present study, we used the right atrium tissue to determine the expression of DGK isoforms. Chronic mitral regurgitation is a state of volume overload that causes complex hemodynamic changes [20-22]. Chronic mitral insufficiency leads to the enlargement of the left atrium, pulmonary congestion, and failure of the right heart. Pulmonary hypertension occurs frequently (in 76% of cases) in patients with isolated chronic mitral regurgitation with preserved left ventricular systolic function [23]. Samples of the left ventricular myocardium obtained from patients who were undergoing orthotopic cardiac transplantation have been used in several studies, thereby suggesting that the hearts were in the state of end-stage in most cases, and were modified by endogenous and exogenous stimuli [24,25]. In the light of these facts, in this study, the right atrium samples were obtained from patients with chronically stressed hearts; these samples were suitable for determining the clinical significance of DGK in modulation of progressive heart failure.

We detected 4 DGK isoforms belonging to 4 different classes in the human heart. Unlike DGKα expression in rodent hearts, DGKγ, another class I DGK, was expressed in the human heart, thereby implying that DGKα in the rodent model can be applied as a molecular target for confirming the clinical significance of DGKγ in the human heart. Although no changes were detected in the expression level of DGKγ in failing heart, we suspected that DGKγ might be activated and might contribute to the process of progressive heart failure. Since the class I DGKs are characterized by the presence of an EF hand motif (a Ca^2+^-binding domain) [26], Ca^2+ ^overload, which is one of the key features of a failing heart and which induces mitochondrial disorganization and cardiomyocyte apoptosis [27], might modulate the activity of class I DGKs in failing hearts.

We identified the expression of DGKη in the human right atrium but could not detect it in rodent hearts [14,28]. Although its functional role is not yet clear, it is noteworthy that the expression of DGKη was increased in the failing hearts affected by volume overload. Recently, Yasuda et al. have reported that DGKη activates Ras/B-Raf/C-Raf/MEK/ERK signaling pathway by regulating B-Raf-C-Raf heterodimer formation [28], thereby suggesting that increased DGKη expression might affect the process of heart failure. Understanding of the role of DGKη in human heart failure might be valuable for determining a novel therapeutic target in the future.

Downregulation of DGKε in rat hearts was observed in both myocardial infarction and aortic banding models [13,14]. In the present study, expression of DGKε was unchanged in the failing human hearts. One possible explanation for this discrepancy is that regulation of DGK isoform expression might be different in different species under different hemodynamic conditions.

In this study, atrial expression of DGKζ, which belongs to class IV, was significantly decreased in the human hearts affected by volume overload. On the other hand, several contradictory findings have reported in animal models of heart failure. In rat hearts affected by chronic pressure overload, translocation of DGKζ from nuclear to cytosolic cell fraction was indicated [13]. DGKζ upregulation was reported in the peripheral zone of the necrotic area in infarcted rat hearts [14]. We have previously reported that DGKζ mRNA levels in neonatal cardiomyocytes increased in the acute phase, but immediately returned to basal levels after endothelin-1 stimulation [15]. In this study, since the hearts were under continuous strain for a long time due to volume overload, DGKζ expression might be decreased in failing human hearts. We have previously reported the importance of DGKζ in abrogating the progress of ventricular remodeling. DGKζ has been reported to inhibit endothelin-1-induced PKCε translocation and hypertrophic responses in neonatal rat cardiomyocytes [15]. Cardiac-specific overexpression of DGKζ has been reported to prevent angiotensin II- and phenylepinephrine-induced activation of several PKCs and subsequent cardiac hypertrophy [16]. Our findings may reflect a pathophysiological importance of DGKζ in the regulation of cardiac hypertrophy and heart failure in the human heart. On the basis of these facts, we thought that upregulation of DGKζ could be a therapeutic target in patients with heart failure.

## Conclusions

In conclusion, this study is the first to provide evidence of differential regulation of human DGK isoforms in failing human heart affected by volume overload, thereby suggesting that individual DGK isoforms may have unique properties, and consequently, distinct functions in the regulation of cardiac hypertrophy and heart failure.

## Competing interests

The authors declare that they have no competing interests.

## Authors' contributions

OB and SS carried out the RNA isolation and RT-PCR. TS evaluated the expressions of DGK isoform and compared those expression patterns with rodent. TS and TK compared the medical record regarding clinical and hemodynamic characteristics of patients with heart failure. ST and MS obtained heart samples from patients. YT and IS conceived of the study and participated in its design and coordination. KG participated in the characterization of the DGK isoforms in human. All authors read and approved the final manuscript.
